# Sulfate handling in proximal renal tubular defects: an exploratory study

**DOI:** 10.1007/s00467-026-07359-7

**Published:** 2026-05-19

**Authors:** Emil den Bakker, Miriam Wamelink, Desiree E. C. Smith, Elena N. Levtchenko, Arend Bökenkamp

**Affiliations:** 1 Department of Pediatric Nephrology, Emma Childrens Hospital, Amsterdam, the Netherlands; 2 Laboratory Genetic Metabolic Diseases, Department of Laboratory Medicine, Amsterdam Gastroenterology Endocrinology and Metabolism (AGEM), Amsterdam , UMC Location University of Amsterdam, Amsterdam, The Netherlands

**Keywords:** Sulfate, Dent disease, Cystinosis, Lowe syndrome

## Abstract

**Background:**

Renal tubular disease causes loss of electrolytes and other molecules. One of these electrolytes is inorganic sulfate, which is reabsorbed by dedicated transporters, including NaS1, encoded by the *SLC13A1* gene. Still, inorganic sulfate is rarely measured in clinical practice, although it is essential for the development and functioning of several organ systems. The importance of adequate sulfate stores is emphasized by genetic disorders impairing sulfate metabolism in the brain, bone, and the endocrine system, causing severe neurodevelopmental disorders, skeletal dysplasia, and hormonal imbalance. Low availability due to renal loss of inorganic sulfate has also been linked to these disorders. We aimed to assess the prevalence of renal inorganic sulfate wasting in renal tubular diseases in a small cohort as an exploratory study.

**Methods:**

Inorganic sulfate was measured in serum and urine of patients with proximal renal tubular disorders using remnant material obtained during routine check-ups. Fractional excretion of inorganic sulfate was calculated alongside plasma inorganic sulfate concentrations and cystatin C–based eGFR.

**Results:**

Fourteen patients were included, in whom 1 to 4 paired measurements of urine and plasma inorganic sulfate were available. Five patients had decreased plasma sulfate on at least one occasion; 13 patients had increased fractional excretion in at least one measurement. In patients with cystinosis, during cysteamine treatment plasma inorganic sulfate levels were often normal despite increased fractional excretion.

**Conclusions:**

Increased inorganic sulfate loss is prevalent in children with proximal tubular defects and sulfate stores can be repleted by oral drugs like cysteamine.

**Graphical abstract:**

A higher resolution version of the Graphical abstract is available as Supplementary Information. Please also add its.pptx as ESM with the name Graphical abstract. Please see https://doi.org/10.1007/s00467-021-05287-2 for reference

**Figure:**
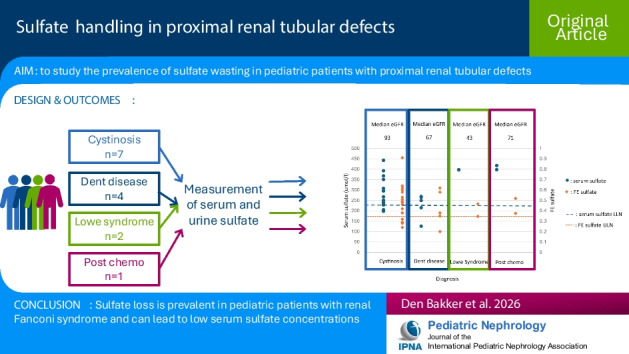

**Supplementary Information:**

The online version contains supplementary material available at 10.1007/s00467-026-07359-7.

## Introduction

Patients with renal tubular diseases lose electrolytes and other molecules into urine. Sodium, potassium, calcium, magnesium, chloride, bicarbonate, and phosphate are regularly measured and supplemented if low. Still, inorganic sulfate, the fourth most abundant anion, is rarely measured in clinical practice, possibly due to pre-analytical and analytical challenges impairing measurement on common automated platforms [1].

Physiologically, sulfate is incorporated into various molecules through the process of sulfonation in which 3′-Phosphoadenosine-5′-phosphosulfate (PAPS) is the final sulfate donor. Proteoglycans, cholesterol, and cholesterol derivatives such as bile acids and steroid hormones, as well as exogenous substances, are sulfonated, which leads to changes in the structure, solubility, and function of these molecules [2]. Genetic variants that impair sulfonation in specific tissues cause neurological developmental disorders [3], distinct forms of skeletal dysplasia [4], hypertension [5], and androgen excess [6]. Low serum levels of inorganic sulfate due to genetic defects in renal inorganic sulfate handling result in similar phenotypes [7, 8].

Inorganic sulfate is filtered in the glomeruli and reabsorbed in the proximal tubules by sodium-sulfate co-transporter 1 encoded by the *SLC13A1* gene [7, 8]. It is likely that patients with a more generalized proximal tubular defect lose inorganic sulfate and have lower sulfate stores similar to patients with specific genetic disorders of renal inorganic sulfate wasting.

This study aims to identify the prevalence and degree of renal inorganic sulfate loss in a cohort of pediatric patients with well-defined proximal renal tubular diseases.

## Methods

This study used cross-sectional and retrospective data of patients with proximal renal tubular disorders. We have set up this exploratory pilot study to assess whether sulfate loss in proximal tubular defects is worth studying in larger cohorts. Inclusion criterion was a proximal renal tubular defect evidenced by increased excretion of low-molecular-weight proteins, phosphate, potassium, bicarbonate and/or glucose. Patients (and/or their caregivers) were approached for consent to use remnant paired blood and urine samples to measure inorganic sulfate and gave written consent. The study was approved by the institutional review board of Amsterdam University Medical Center (number, 2025.0531). Inclusions and measurements occurred on a convenience basis between January 2024 and October 2025, meaning that patients with more frequent visits in that time period could have more than one measurement. Additional information, such as primary diagnosis, serum and urine creatinine and serum cystatin C levels, medication, height and weight were taken from the patient charts. We specifically recorded cysteamine medication as this drug contains a thiol moiety and is therefore a potential sulfate supplement.

Serum and urine inorganic sulfate were measured using a Sciex API5000 liquid chromatography–tandem mass spectrometer using negative electrospray ionization. Serum was deproteinated before measurement using acetonitrile [7].

Serum and urine creatinine were measured using an IDMS traceable enzymatic method, serum cystatin C with an IFCC-traceable nephelometric immune-assay.

Glomerular filtration rate (GFR) was estimated using the cystatin C–based full age spectrum (FAScys) equation [9]. Fractional excretion (FE) of inorganic sulfate was calculated as follows:

$$FE=(urine\;sulfate\;(\mu mol/L)\;\times\;serum\;creatinine\;(\mu mol/L))/(serum\;sulfate\;(\mu mol/L)\;\times\;urine\;creatinine\;(mmol/L)\;\times\;1000)$$

Normal values for serum inorganic sulfate (231–572 µmol/l) were determined in our laboratory based on 52 control subjects (age, 0–80 years), which had very similar results to those described in the literature [10, 11]. No age dependency was noted within the control samples. The fractional excretion of inorganic sulfate (0.17–0.34) was taken from the study by Bowling et al. [11].

## Results

**Fig. 1:**
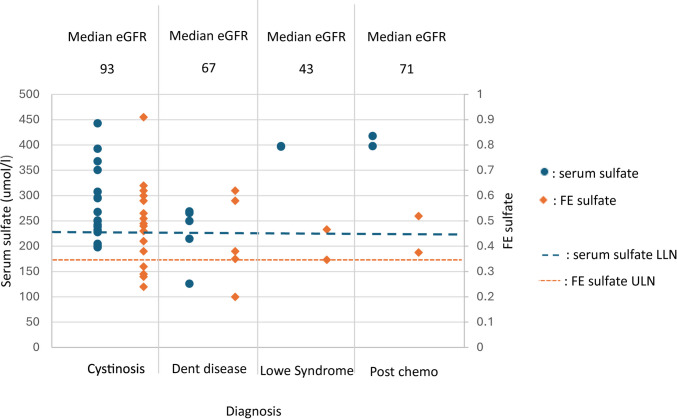
Serum sulfate concentration and FE sulfate grouped by diagnosis. eGFR, estimated glomerular filtration rate; FE, fractional excretion; LLN, lower level of normal; ULN, upper level of normal

We included 14 individual patients from whom at least one paired measurement of serum and urine inorganic sulfate was available. Paired urine and blood samples were obtained within a maximum of 2 h. Results are summarized in Table 1. Primary diagnoses included cystinosis (*n* = 7; 17 measurements), Dent disease (*n* = 4; 5 measurements), Lowe syndrome (*n* = 2; 2 measurements), and post-chemotherapeutic tubulopathy (*n* = 1; 2 measurements). Median serum inorganic sulfate differed between diagnosis groups (Fig. 1). In patients with cystinosis, the median serum inorganic sulfate was 269 µmol/l (IQR, 236–339), in Dent disease 250 µmol/l (IQR, 170–267), in Lowe syndrome 397.5 µmol/l (IQR, not applicable), and in the patient with post-chemotherapy tubulopathy, 398 µmol/l (IQR: not applicable). Using independent-samples Kruskall–Wallis testing, we found significantly lower median serum inorganic sulfate in Dent disease patients compared to Lowe syndrome (*p*, 0.014) and post-chemotherapy tubulopathy (*p*, 0.015), but not cystinosis patients (*p*, 0.185). In 5 out of 14 patients (36%), at least one inorganic sulfate measurement was below the reference range. In 13 out of 14 patients (93%), the fractional excretion of inorganic sulfate exceeded the reference range on at least one occasion. Cysteamine was given to all patients with cystinosis except for patient C-6, in whom the first measurement (C-6–1) was performed at the time of diagnosis before initiation of cysteamine treatment. On 4 out of 5 occasions, the patients with Dent disease had elevated FE sulfate, while serum inorganic sulfate was borderline low or decreased in all (Fig. 1). Patient D-1 initially presented with a skeletal phenotype described as metaphyseal dysplasia along with rickets, which only partially responded to phosphate supplementation. His height still is below his target height range.

**Table 1:** Descriptive results from our cohort of patients with renal Fanconi syndrome

Patient	Primary diagnosis	Age (years)	Serum sulfate (umol/l)	FE sulfate	Cysteamine dose (mg/m^2^/day)	eGFR cystatin C (ml/min/1.73 m^2^)
C-1–1	Cystinosis	5.25	250	0.28	1250	101
C-1–2	Cystinosis	5.42	228^$^	0.24	1184	n.a
C-1–3	Cystinosis	5.67	198^$^	0.32	1184	96
C-1–4	Cystinosis	6.25	251	0.58*	1363	96
C-2–1	Cystinosis	2	242	0.62*	803	n.a
C-3–1	Cystinosis	18.6	393	0.91*	1087	n.a
C-4–1	Cystinosis	12.6	268	0.51*	588	102
C-4–2	Cystinosis	13.3	239	0.29	665	96
C-4–3	Cystinosis	13.6	201^$^	0.53*	779	113
C-5–1	Cystinosis	7.9	296	0.60*	1050	90
C-5–2	Cystinosis	8.3	308	0.64*	1200	76
C-5–3	Cystinosis	8.9	351	0.42*	1350	76
C-6–1	Cystinosis	1.7	205^$^	0.42*	0	52
C-6–2	Cystinosis	1.8	368	0.49*	1429	48
C-6–3	Cystinosis	2.2	443	0.46*	1333	50
C-7–1	Cystinosis	3.5	232	0.48*	1406	111
C-7–2	Cystinosis	3.8	295	0.46*	1384	101
D-1–1	Dent	8.4	126^$^	0.62*	0	66
D-2–1	Dent	11.25	215^$^	0.38*	0	73
D-3–1	Dent	4.6	266	0.35*	0	78
D-3–2	Dent	5.1	250	0.58*	0	87
D-4–1	Dent	15	269	0.20	0	68
L-1–1	Lowe	12.5	398	0.35*	0	42
L-2–1	Lowe	14.9	397	0.47*	0	44
O-1–1	Post chemo	17.1	418	0.52*	0	68
O-1–2	Post chemo	17.6	398	0.38*	0	74

*n.a.* not available

^$^Serum sulfate measurements below the lower limit of normal [*N* = 231–572 µmol/l]

^*^Fractional excretion (FE) exceeding the upper level of normal [*N* = 0.17–0.34]

## Discussion

We show that low serum inorganic sulfate and high FE of inorganic sulfate are prevalent in pediatric patients with renal Fanconi syndrome. This is in line with a previous report suggesting lower stores of inorganic sulfate in these patients [12]. Still, the primary reference cited in this paper was only published in abstract form. While low inorganic sulfate levels were most pronounced in patients with Dent disease, most patients with cystinosis showed normal serum levels of inorganic sulfate despite a high fractional excretion. We suspect that this is due to the high sulfur content of cysteamine (13 mmol/g), which can act as an inorganic sulfate precursor, either directly through the taurine pathway [13] or indirectly through increasing cysteine concentration in cells and thereby providing sulfate through the cysteine–sulfinate pathway [14, 15]. Of note, cystinosis patient C-6 had a very low inorganic sulfate level and a high fractional excretion before starting cysteamine treatment. In line with our hypothesis, serum inorganic sulfate concentrations normalized and the fractional excretion increased even more upon cysteamine treatment. Interestingly, patient C-1 had consistently low serum inorganic sulfate in the absence of a high FE sulfate on 3 out of 4 occasions. It also appears that serum inorganic sulfate concentrations do not seem to be correlated with cysteamine dose suggesting that there is some variation in the effectiveness of inorganic sulfate generation from cysteamine or with the timing of sampling in relation to the administration of cysteamine. It should be borne in mind that about 40% of sulfate intake occurs in its inorganic form, particularly from drinking water, in which the sulfate content is highly variable [16]. The remainder comes from animal proteins rich in methionine and cysteine [16]. Also, there is significant recycling of sulfate-containing compounds involving PAPS to ensure sufficient sulfate availability [2].

Inorganic sulfate clearance is directly related to inulin clearance and patients with severely decreased GFR below 30 ml/min/1.73 m^2^ have increased serum levels of inorganic sulfate [17]. Also, there is an inverse relationship between FE inorganic sulfate and inulin clearance [17]. This fits with our observation that the two patients with Lowe syndrome, who have the lowest eGFR in our cohort, have the highest serum levels despite having an increased FE sulfate.

Due to variance of diagnoses, sulfate supplementation from cysteamine and eGFR in our small cohort, we were unable to perform statistical tests to correlate sulfate levels to clinical outcomes that might be related to low sulfate stores such as height, blood pressure, developmental, or endocrine disorders. However, patient D-1, who has the lowest serum inorganic sulfate level, is growth retarded compared to his target height range and presented with skeletal abnormalities, which persisted after correction of hypophosphatemia. He ultimately required orthopedic surgery.

Further research should aim to create a larger, more homogeneous cohort of individuals with a proximal tubulopathy to correlate inorganic sulfate handling with outcome measures. We suggest starting with a cohort of Dent disease patients as they have the fewest features of extra-renal disease, higher eGFR and do not receive cysteamine as a potential sulfate precursor. Considering the normalization of serum concentrations with cysteamine, further studies should investigate if this can also be achieved with less costly and less toxic over-the-counter drugs like acetylcysteine.

## Conclusion

Inorganic sulfate loss seems to be a feature of renal Fanconi syndrome in pediatric patients and can lead to low serum inorganic sulfate concentrations. This can in part be restored with the sulfur-containing drug cysteamine, which forms the mainstay of cystinosis therapy. The clinical relevance of these findings as well as the prevalence of this feature requires further study.

## Supplementary Information

Below is the link to the electronic supplementary material.

Graphical abstract (PDF 197 KB)

ESM2 (DOCX 35.2 KB)
