# Supplementary material for: Sulfate handling in proximal renal tubular defects: an exploratory study
Source: Pediatr Nephrol. 2026 May 19;41(10):3605–9. doi: 10.1007/s00467-026-07359-7 (PMC13481404; doi:10.1007/s00467-026-07359-7)
Supplement: Supplementary file 1 [file 467_2026_7359_MOESM1_ESM.pdf]

Sulfate handling in proximal renal tubular defects

Original Article

AIM: to study the prevalence of sulfate wasting in pediatric patients with proximal renal tubular defects

DESIGN & OUTCOMES:

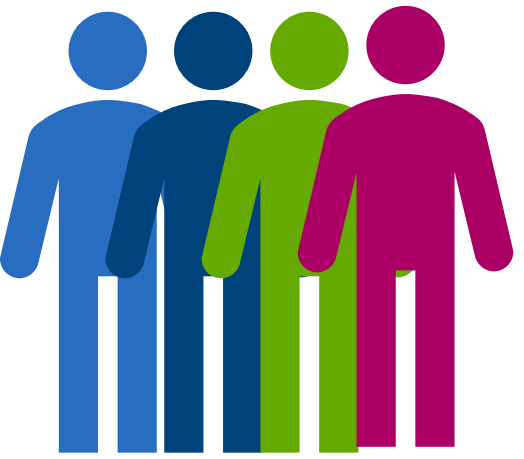

Cystinosis n=7

Dent disease n=4

Lowe syndrome n=2

Post chemo n=1

Measurement of serum and urine sulfate

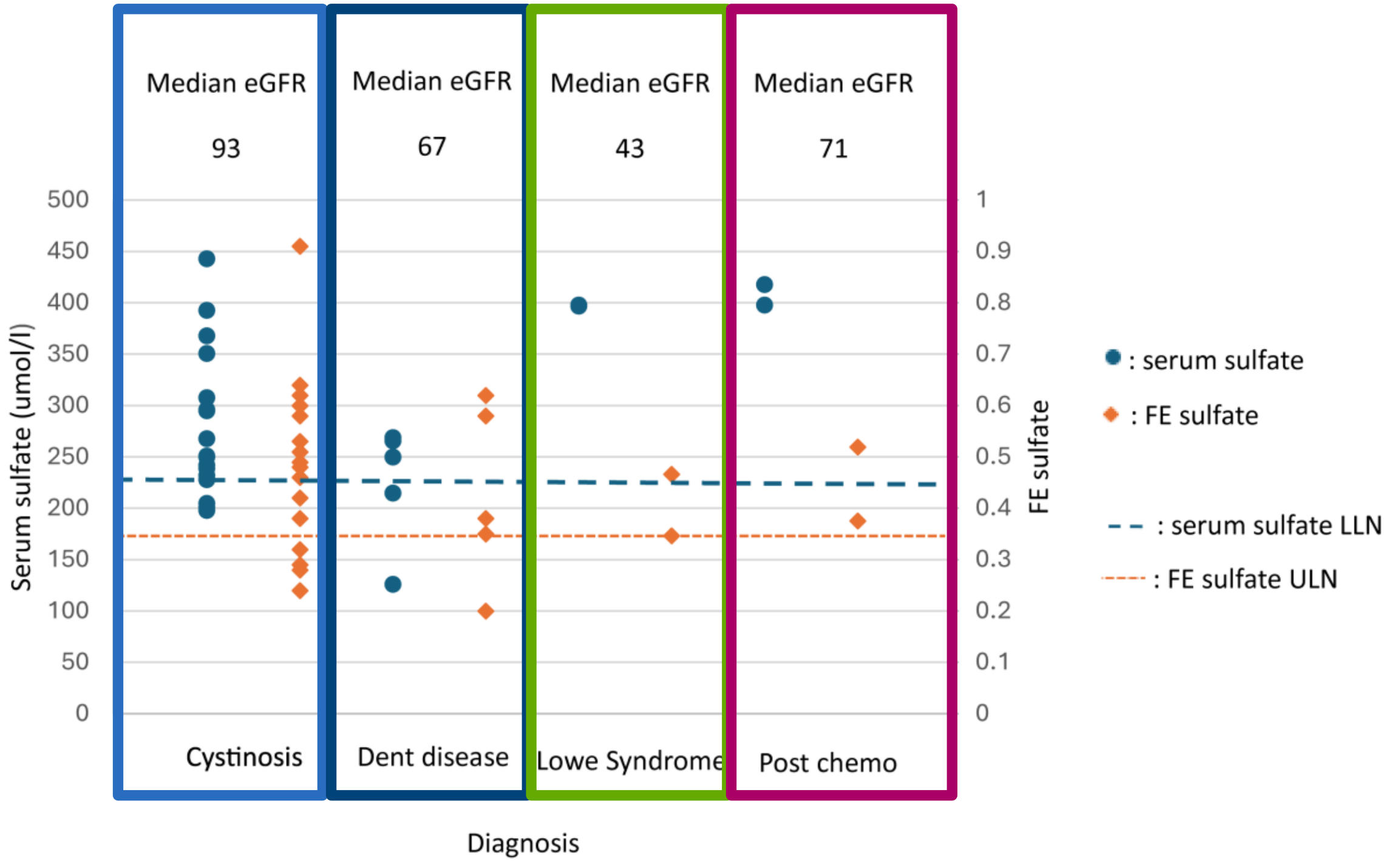

CONCLUSION: Sulfate loss is prevalent in pediatric patients with renal Fanconi syndrome and can lead to low serum sulfate concentrations

Den Bakker et al. 2026

Pediatric Nephrology
Journal of the
IPNA International Pediatric Nephrology Association
